# Mental health education on college students’ english vocabulary memorization from the perspective of STEAM education

**DOI:** 10.3389/fpsyg.2022.944465

**Published:** 2022-09-30

**Authors:** Yu Wang

**Affiliations:** School of Foreign Languages, Shaanxi Xueqian Normal University, Xi’an, China

**Keywords:** English vocabulary memorization, mental health education, teaching model, psychology, influencing factors

## Abstract

The formation and research of Constructivism theory is another understanding in developing educational psychology. Many problems exist in English vocabulary teaching and learning, which are too common to be noticed, negatively and implicitly impacting Students’ English Vocabulary Memorization (EVM). In order to solve these problems, this work studies college Students’ EVM from the perspective of Constructivism. Firstly, the literature review and observation method understand the Students’ EVM performance and teaching behavior. Secondly, it analyzes the current situation, problems, and reasons for Students’ poor EVM abilities. Finally, combined with relevant theories, a college Students’ EVM-oriented teaching model is proposed, whose effectiveness is verified by teaching experiments. The experiment recruits100 freshmen, including 60 boys and 40 girls, and lasts half a semester. The average score of the final exam in the experimental group is more than 90, which is better than that in the control group. Then, the SPSS21.0 is used in the independent-samples *t*-test, finding no significant difference (*P* < 0.05). Thus, the experimental group’s comprehensive English Proficiency Level (EPL) has not been negatively affected. The proposed teaching model can improve Students’ EVM efficiency and consolidate their memory. The proposal has important guiding significance for stimulating Students’ interest in English vocabulary learning and the quality of vocabulary teaching.

## Introduction

Psychology can deepen the significance of pedagogy and educational psychology through cognitive understanding. The formation and research of constructivism theory is a new perspective of educational psychology development. Stimulating Students’ interest in learning English and improving the quality of vocabulary teaching are still important issues in the current education and teaching environment. Language research mainly involves pronunciation, vocabulary, and grammar. In particular, vocabulary is the basic language unit with meaning. In English learning, vocabulary affects learners’ English Proficiency Level (EPL) in listening, speaking, reading, writing, translation, and communication. Therefore, the importance of vocabulary can never be overemphasized in English teaching. Concerning the current situation of English vocabulary teaching, Colleges and Universities (CAUs) need to explore effective and innovative learning strategies to improve learners’ comprehensive EPL and oral communication. Constructivist learning theory holds that learning is a process of actively constructing internal psychological representation. Unlike the traditional knowledge memorizing approach, constructive learners acquire and construct knowledge through interaction with the outside using the original experience. It is more effective than the “stuffing” teaching method in Students’ vocabulary learning. Mental Health Education (MHE) is based on Students’ physiological and psychological development laws. It integrates psychological approaches into education to cultivate Students’ positive psychology and overall quality (Yang Q. F. et al., 2020).

STEAM education originated in the United States in the 1990s. It is the extension of STEM: Science, Technology, Engineering, and Mathematics by considering creativity in practice. That is, it adds Art to STEM education. STEM education is an educational theory and practice aimed at cultivating innovative talents. The core idea of STEAM education includes five aspects: (1) The basic concept of student-centeredness; (2) Interdisciplinary learning content; (3) Learning activity form based on project/problem/design; (4) Interactive form of communication and expression; (5) Innovative achievement-oriented learning objectives. It aims to enable learners to develop problem-solving, communication, cooperation, expression ability, and innovation literacy in experience and creation. Currently, MHE is mainly the form of psychological counseling; other than being a compulsory subject, it only supplements the main disciplines (Zhang et al., 2022). Teachers consciously use psychological theories and technologies to improve Students’ cognitive, emotional, and behavioral levels and Students’ learning motivation and memory skills (Yang et al., 2018; Wise et al., 2021). Fusing MHE with other disciplines can improve college Students’ psychological wellbeing, learning potential, and overall development.

Most language learners are frustrated with vocabulary memorization, which might be the biggest obstacle to learning (Pratt et al., 2021). The cause might be explained from two dimensions. Firstly, there is a lack of in-depth thinking on what they are trying to remember. Secondly, most students only mechanically repeat words without learning interest, making it hard to persist (Zhai, 2021). As a result, Students’ EPL cannot meet teachers’ expectations, Students’ learning interest declines, and initiative elapses (Khan and Liu, 2020). Not surprisingly, reading and speaking become increasingly difficult as they proceed to higher grades. New words become extremely difficult to remember, and old ones are often forgotten (Karousou and Nerantzaki, 2020).

Through observation and investigation in practice, this work confirms the underlying reasons for college Students’ poor English Vocabulary Memorization (EVM) ability as the disconnection between theories and practice. It applies the forgetting curve theory. Besides, Students’ intelligence factors (memory, attention, observation, imagination, and thinking) and non-intellectual factors (motivation, interest, will, emotion, and personality) related to learning have not been given full play in the practice of learning and memorizing vocabulary. Analyzing and solving these problems is of great practical and theoretical significance in college English teaching. In particular, school MHE can be infiltrated into college English teaching to provide a possible solution to college Students’ EVM through more scientific and effective means.

The aim is to reduce Students’ English learning pressure and help them learn vocabulary more effectively. There were corresponding Constructivist theories in the early thought of educational psychology. However, they did not form a certain ideological scale. Therefore, Constructivism in educational psychology develops and progresses in constant exploration and then reaches the current state. Based on Constructivist Psychology, this work puts forward some EVM strategies to assist teaching and learning. Firstly, literature review and observation methods understand the EVM performance, learning behaviors, and the reasons behind the poor EVM. Secondly, combined with the relevant theories, it puts forward the college Students’ EVM-oriented teaching model and verifies its effectiveness through teaching experiments. Finally, the factors influencing EVM of college students are analyzed through a Questionnaire Survey (QS). The QS data are compared and analyzed to improve the model. The innovation lies in introducing MHE into the college Students’ EVM based on Participant Action Research (PAR).

**FIGURE 1:**
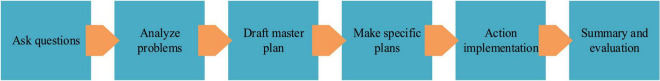
Design of PAR.

The design of PAR is shown in Figure 1. Studying vocabulary teaching can help teachers make up for their shortcomings, catch up on the latest teaching techniques, and formulate appropriate teaching strategies. It also enables teachers and students to be aware of vocabulary’s important role. Meanwhile, it changes old teaching and learning ideas, solves problems in vocabulary teaching, and helps them regain enthusiasm and confidence in English teaching and learning. Finally, it achieves the goal of improving Students’ comprehensive EPL.

## Research on english vocabulary memorization

Indeed, EVM is boring as other fundamental skill acquisition in English learning. Hence, enriching EVM strategies and stimulating Students’ interests are key to English educational reform. Yue (2017) studied Computer-Aided Design (CAD)-based multimedia English vocabulary teaching courseware and summarized the CAD multimedia technological concepts, characteristics, development, and application status. He analyzed the cognitive learning theory and memory law. The English vocabulary teaching courseware’s module, style, and database were designed based on scientific guidance, demand analysis, and model construction. According to subjective and objective evaluations, the experimental results showed excellent application effects (Yue, 2017). Bai (2018) defined vocabulary learning as mastering a certain number of words or phrases and their usage. Correct strategies were critical, and there must be customized methods for every language learner to help them learn vocabulary independently (Bai, 2018). Andrä et al. (2020) studied the effects of hands-gesture-based and picture-based learning on 8-year-olds’ vocabulary acquisition skills in German. Children were taught five consecutive days of English vocabulary, concrete and abstract. Experiments 1 and 2 used “gesture reinforcement” (children listen to an English word and represent it with a specific gesture) compared with non-reinforcement baseline conditions. By comparison, Experiment 3 has compared gesture reinforcement with “picture reinforcement” (children look at a picture and guess an English word). Then, children were tested for vocabulary recall and translation 3 days, 2 and 6 months after learning. Compared with non-reinforcement learning, gesture and picture reinforcement could improve children’s test scores. The benefits of rich gestures and pictures lasted 6 months after training in both concrete and abstract vocabulary. The results showed that both gestures and pictures could improve children’s performance in second language learning. Besides, long-term academic performance benefits were also very high (Andrä et al., 2020).

**FIGURE 2:**
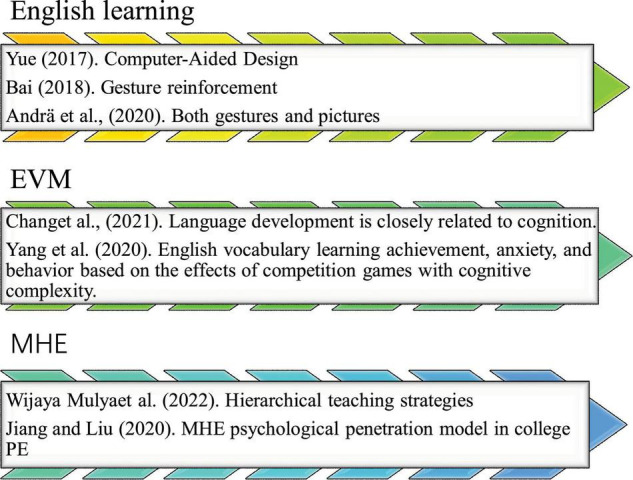
Key research of EVM.

Chang et al. (2021) studied EVM from a cognitive perspective. As an advanced cognitive function, EVM was closely related to language development and cognition. Cognitive research provided a theoretical basis and methodology for language development and learning key research of EVM is shown in Figure 2. In this field, metalanguage is mainly studied along with phonological orthography and morphological awareness. These theories reflected vocabulary learning and literary development. The final results showed that vocabulary learning based on cognitive linguistics could effectively change English teaching practice (Chang et al., 2021).

Some studies have shown that language learning based on digital games can promote learning motivation and immersive learning. However, some game elements (e.g., competition and challenges) or learning contents (e.g., difficulty level) may have distinct effects on different learners. Especially, competitive games may lead to frustration for those with low self-efficacy or academic achievement. Therefore, a competitive learning environment must consider Students’ cognitive ability and learning content complexity. Yang X. H. et al. (2020) studied the English vocabulary learning achievement, anxiety, and behavior based on the effects of competition games with cognitive complexity. A game-based situational vocabulary learning system was developed by integrating competitive strategies based on cognitive complexity to provide learners with appropriate tasks. An experiment was conducted in a senior high school English course to evaluate the model’s effectiveness. It was found that, compared with traditional situational game methods, situational games using competitive strategies based on cognitive complexity significantly improved participants’ academic performance (especially students with poor academic performance) and compounded their anxiety (Yang X. H. et al., 2020). Additionally, the behavioral analysis showed that students could complete the tasks more smoothly using this method because the system considered the player’s learning performance and cognitive complexity. It upgraded or demoted the learner’s game level to ensure students learn appropriately (Susanto, 2017).

MHE is rich with content and can combine with various subjects. In classroom teaching, teachers should consider Students’ intellectual and non-intellectual factors. Learning strategies can be cultivated to develop Students’ learning potential. At the same time, teachers should improve Students’ learning efficiency and cultivate lifelong learning habits (Liu, 2021). Hierarchical teaching strategies can be employed to create a harmonious classroom atmosphere and the teacher-student relationship. Cultivating Students’ listening, homeworking, previewing, and resting habits helps students adapt to the learning environment and understand the learning tasks. On the other hand, students should be guided to master examination skills and adjust their examination mentality. Learning weariness and polarization must be prevented, as well as criticism and punishment-based educational means and evaluation. There is a need to release learning burdens further and encourage targeted educational modes that stress initiative and creativity. As such, students can find a sense of achievement, joy, fun, and enthusiasm in learning (Wijaya Mulya et al., 2022). Many CAUs have tried to combine MHE with Physical Education (PE) to improve the effectiveness of PE. Jiang and Liu (2020) designed an MHE psychological penetration model in college PE with three stages: interaction, classification, and evaluation. Then, the model was randomly applied to Students’ PE teaching in four universities. Consequently, student acceptance, teacher satisfaction, and student satisfaction indexes were selected to evaluate the teaching effect, classroom influencing factors, and mental health influencing factors. The results showed that 72% of the subjects had a high understanding of the penetration model. 70% of the subjects were most concerned about teaching style and teaching fun. The mental health status of all subjects has been improved to varying degrees.

To sum up, EVM is the key to English learning. There is extensive research on English vocabulary teaching and memory, such as cultivating vocabulary memory skills, classified memory, the forgetting law, and context-based approaches. Each has certain advantages but is affected by subjective and objective factors; these methods are somehow divorced from the current English teaching and learning. There are still few studies on subject integration: for teachers to be both an instructor and a researcher is very challenging. However, it provides a broad stage for teachers to display their talents. Therefore, this work uses the PAR method to study the impact of MHE on college Students’ EVM.

## Common problems of college students’ english vocabulary memorization and their causes

**FIGURE 3:**
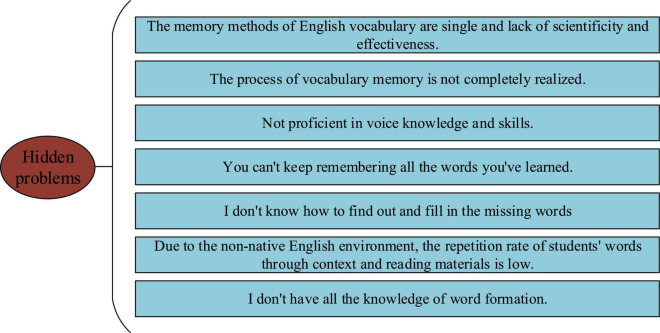
Problems in college Students’ EVM.

It is observed that there are many problems in college Students’ EVM, which are too common for students to notice even if they have a great impact. Figure 3 illustrates seven types of common problems obtained through analysis.

The above problems are analyzed to reveal the inner causes of poor English vocabulary teaching and learning situations to find new methods.

### Causes from students’ perspective

Three closely related reasons might explain the common problems in college Students’ EVM: learning methods, knowledge base, and psychological factors. Firstly, lousy learning methods will reduce learning efficiency and interest, leading to learning weariness, and impairing Students’ ability to absorb knowledge (Contractor et al., 2020; Murphy et al., 2021). Besides, a lousy learning method might fail to teach students the habit of learning throughout their lives or fully exert their potential in creativity or initiative (Chen et al., 2019). In the long run, students will lose the sense of achievement in English learning and give up memorizing vocabulary (Tiing et al., 2021).

Secondly, pronunciation and spelling are the foundation of memorization. Students without proper knowledge of syllables will have difficulty memorizing words. Therefore, the knowledge base is another factor underlying low memory efficiency (Noprianto and Purnawarman, 2019). Lastly, psychological factors, such as lack of interest, weak self-discipline, and emotional instability, significantly impact college Students’ EVM. Therefore, it is necessary to infiltrate MHE into college Students’ EVM by exerting the positive role of Students’ psychological factors in learning.

### Causes from the college educational perspective

Unlike middle school students, undergraduates are given more free time. Without supervision, some might not be as active as before in learning. Besides, college courses are much more difficult and demand excessive time and effort. These factors all squeeze Students’ time for EVM and extra curriculum reading in English. Therefore, the context-based EVM approach might not be ideal in CAUs (Akdogan, 2017). Moreover, English teachers follow the traditional teaching methods, and Students’ EVM method is single (Ansarin and Khabbazi, 2021). Besides, English teachers lack in-depth exploration and research into the teaching methods. Thus, the current teaching mode cannot stimulate Students’ learning motivation, enthusiasm, sense of achievement, and confidence.

### Causes from the teaching methods’ perspective

**FIGURE 4:**
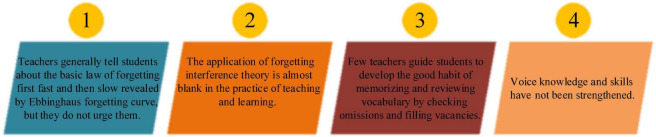
Causes from the college English vocabulary teaching methods’ perspective.

Literature review unveils that the English vocabulary teaching methods are monotonous in most CAUs and less scientific or practical. Hence, teachers cannot cultivate Students’ interest in learning, arousing Students’ negative psychologies, hindering memorization efficiency, and causing other problems (Wu and Huang, 2017). Many techniques can be chosen in EVM, including repeated reading, associative memory, and focused word recognition. However, there is still no practical college students-oriented EVM method (Hao et al., 2019). On top of the traditional teaching methods, some teachers are trying new ones. However, the new methods are only effective when students can use vocabulary flexibly. Hence, most new explorations result in premature death. Figure 4 exemplifies the main reasons from four aspects.

**FIGURE 5:**
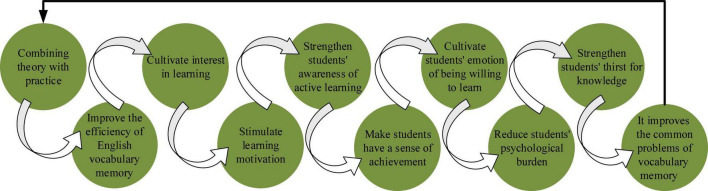
Benign cycle of EVM.

**FIGURE 6:**
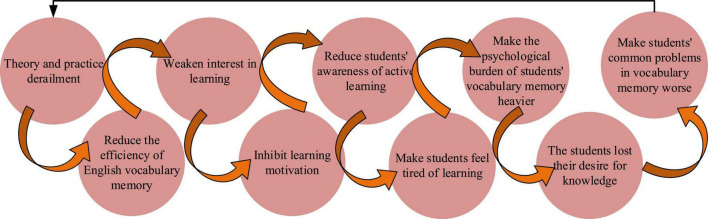
Vicious circle of EVM.

In short, the disconnection between the psychological theory and practice of EVM is the fundamental reason for the ineffective English vocabulary teaching. A well-connected theory and practice are reciprocal. Psychological factors have two main roles in EVM: the benign cycle (Figure 5) and the vicious cycle (Figure 6).

### Infiltration of mental health education into students’ english vocabulary memorization

MHE can permeate other subjects with extremely rich content. In order to do so, teachers should fully consider Students’ intellectual and non-intellectual factors in their teaching links. Meanwhile, they should train learning strategies, improve learning methods, and develop Students’ learning potential. At the same time, learning efficiency must be improved to cultivate students with lifelong learning habits. On the other hand, the infiltration of MHE promotes hierarchical teaching. It creates a harmonious classroom atmosphere and the teacher-student relationship. It provides learning guidance and improves learning problems. MHE infiltration can help students form good habits in listening, homeworking, previewing, reviewing, and resting. It helps students adapt to the learning environment and understand learning objectives. Lastly, it guides students to master examination skills and adjust their examination mentality.

In EVM, in addition to stimulating, transforming, and maintaining learning motivation, there is a need to use “Yerkes-Dodson’s law effectively.” This law reveals the relationship between job efficiency and motivation level. The former increases with the latter’s increase until to the optimal level and then decreases. Generally, the operation efficiency is the highest under the moderate motivation level. However, this moderate motivation level is transferred by the complexity of the operation. The simpler the operation is, the higher the moderate motivation level should be. The more complex the job is, the lower the level of this moderate motivation should be. The best learning state occurs when the body is relaxed and the individual is focused. Body relaxation leads to psychological relaxation when the brain is the most open and accessible to absorb external information. That is, students should have a comfortable state of mind when memorizing vocabulary.

## English teaching model based on constructivism

Constructivism emphasizes the relativity, dynamic, and situational nature of knowledge itself. Knowledge cannot reflect reality objectively and purely. Human beings create knowledge, and knowledge is affected by human culture and values. Individuals have different experiences and come from distinct backgrounds, so they also have differences in understanding knowledge. Remarkably, the degree and process of meaning construction vary in different situations. Learners’ minds are not empty boxes. Students’ original knowledge and experience cannot be ignored in teaching. Knowledge should never be imparted to students forcefully and mechanically. Instead, they should be guided to transfer existing knowledge and experience to develop new knowledge. Moreover, educators play the role of assistant and supporter of Students’ knowledge construction and become the cooperator and senior partners of students in knowledge acquisition.

It is very beneficial for learners to use their original knowledge to solve English education and teaching problems. Learners can also deepen their understanding and mastery of knowledge by solving new problems using old knowledge. Therefore, the problem-based teaching model is very beneficial in stimulating Students’ enthusiasm for learning English. This teaching model takes problems as the core and puts knowledge in problem situations to stimulate Students’ imagination and thirst for knowledge. Modern Constructivism holds that learning is not just a process of transferring knowledge but a process in which students actively and consciously build new knowledge. In this process, teachers only play the role of supporters and helpers, and the main body of knowledge acquisition and mastery is students. Constructivism theory points out that even some starters are never a blank sheet. The construction of new experience and knowledge must be developed based on their actual knowledge and experience. Understanding and mastering new knowledge needs the interaction between students and students, teachers and students, and students and teaching elements. English teaching should create an excellent cooperative learning environment and actively encourage students and teachers’ cooperative learning and interactive teaching.

## Solutions to college students’ english vocabulary memorization

**FIGURE 7:**
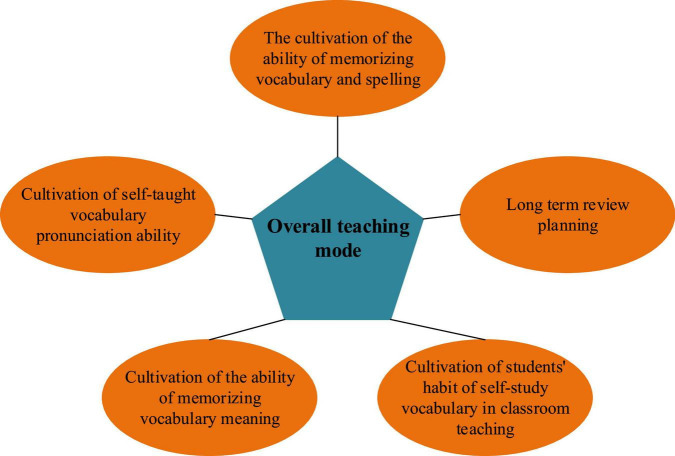
Contents of the proposed teaching model.

Based on the above problem analysis, a college Student’s EVM-oriented teaching model is implemented, as detailed in Figure 7.

The proposed college Students’ EVM-oriented teaching model combines cognitive psychology with vocabulary teaching. It factors in Students’ instantaneous, short-term, long-term, and psychological memory.

### Modeling college students’ english vocabulary memorization teaching

The proposed model starts from Students’ phonetic knowledge: pronunciation to help them read new words correctly, even without teachers’ guidance or other electric terminals. Specifically, it summarizes the letter combination rules in phonetics and syllables. Students can master the spelling rules quickly to prepare themselves for EVM.

**FIGURE 8:**
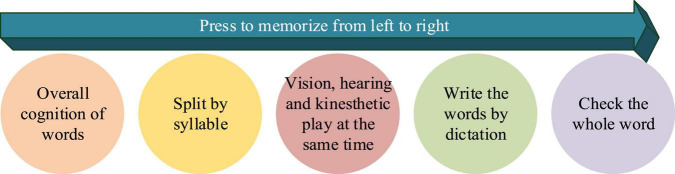
Diagram of word cracking memory method.

Secondly, it cultivates Students’ ability to memorize vocabulary and spelling. Initially, the words are classified according to syllables and then connected. Then, students can remember vocabulary by the method in Figure 8 through sensory perception to improve EVM efficiency.

Thirdly, it cultivates Students’ ability to understand the word’s meaning. To this end, centralized memorization is designed, which can be completed through the syllabus vocabulary (Montoya et al., 2019). This work improves vocabulary usage through the idea of “trying to recall.” Specifically, students recite the vocabulary several times, during which questions are inserted at a random interval for students to recall either the Chinese or the English meanings. The whole process will record Students’ difficulty points for teachers to formulate targeted trading. Additionally, it is observed that the recitation time has a positive linear relationship with the EVM.

Fourthly, teachers are required to cultivate Students’ self-learning habits in classroom teaching as organizers and instructors, fully playing Students’ learning initiative and allowing more time for EVM. Essentially, it is a way of making better use of classroom time. Teachers observe the Students’ self-learning process and guide those who need help throughout the process.

Fifthly, the review must not be overlooked according to the forgetting curve, regression, and interference theories. Methods such as classification and context can also improve EVM efficiency.

**FIGURE 9:**
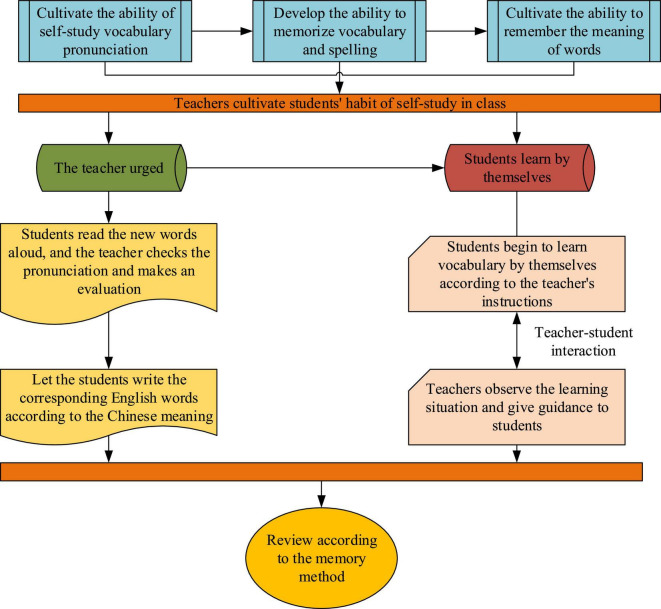
Flow of the proposed teaching model.

Figure 9 signifies the flow of the proposed college Students’ EVM-oriented teaching model.

From Figure 9, the first three steps are the preparation of the last two steps in EVM. Therefore, the following research will focus on the last two steps. This work believes that Students’ EVM can be improved under the proposed teaching model. Increased vocabulary and fluency in reading and speaking raise Students’ learning interest.

### The case verification of the proposed college students’ english vocabulary memorization-oriented teaching model

Here, 100 freshmen are randomly selected from a university in Xi’an, including 60 boys and 40 girls, with an experimental duration of half a semester. They are randomly divided into experimental and control groups, with 50 people in each group. The proposed teaching model is used in the experimental group; then, the results are compared with the control group to test the model’s effectiveness. Before the experiment, Students’ EPL is tested. The new words of the first four units (Unit1, Unit2, Unit3, and Unit4) in College English are selected as the teaching content. The experimental group employs the proposed model, and the control group uses the traditional method. At the end of every unit, students are given a vocabulary test. No review time is given, and Students’ scores are averaged. The purpose is to test the EVM rate after learning one unit in more than 1 week. The EVM rate of the previous units is also tested over time. Importantly, difficult-to-remember words are selected for testing. Each test selects 50 words, 1 point for each word, with a total score of 50 points. Only the part of speech and Chinese meaning required by the syllabus is given. The students are asked to write the corresponding English words within the specified time. Afterward, the test sheet is made anonymous to avoid cheating. The specific test settings are listed in Table 1. Altogether, eight tests are fired, in which Test 1, Test 3, Test 5, and Test 7 correspond to Unit 1, Unit 2, Unit 3, and Unit 4, respectively. Test 2, Test 4, Test 6, and Test 8 test the EVM rate of previously learned units (Test 2 corresponds to Unit 1 after 2 weeks or the 13th week of the semester). The test content is repeated without any change to previous tests. Similarly, Test 4 corresponds to Unit 2 after 2 weeks (the 15th week of the semester). Test 6 is the mixed test of Units 1 and 2 in the 17th week, and Test 8 is the mixed test of Units 1, 2, 3, and 4 in the 19th week. Statistical Package for the Social Science (SPSS) 21.0 is utilized for the independent sample *t*-test on the eight rounds of test results.

**TABLE 1:** The specific test settings.

Number of tests	Test content
Test 1	Vocabulary test in Unit 1
Test 2	Vocabulary test in Unit 1 after 2 weeks
Test 3	Vocabulary test in Unit 2
Test 4	Vocabulary test in Unit 2 after 2 weeks
Test 5	Vocabulary test in Unit 3
Test 6	Mixed vocabulary test in Unit 1 and Unit 2
Test 7	Vocabulary test in Unit 4
Test 8	Mixed vocabulary test in Units 1, 2, 3, and 4

## Results

PAR forms a specific and effective solution after repeated demonstration, experiment, and modification. Learning Students’ unique features and the actual situation of College English teaching are the basis for solving problems in college Students’ EVM. Meanwhile, the previous theories and methods must be integrated into the new teaching model and be applied to long-term teaching practice by infiltrating MHE into English EVM teaching. In order to ensure scientificity, this work consults the literature, analyzes the theories of English vocabulary learning, teaching, and memory, and demonstrates the necessity of psychological factors in learning to play a positive role in EVM. It establishes a new teaching model to solve the problems in college Students’ EVM. The effectiveness of the model is verified from two aspects. (1). Experimental teaching verification. The experimental group and the control group are set up. The experimental group is taught with the proposed teaching model. In contrast, the control group carries out teaching in the traditional way. The test results are compared between the two groups. (2). EPL verification. Language learning develops comprehensive abilities to listen, speak, read, and write based on vocabulary. Relevant theories point out that improving vocabulary will promote comprehensive EPL, but sometimes there will be negative effects. For example, some students focus solely on vocabulary while ignoring other aspects of language learning, resulting in the decline of comprehensive EPL. Such being the case, the Student’s scores for the mid-term and final examinations are both counted and comparatively analyzed as the post-test scores of the experiment.

### Comparison of english proficiency level before the experiment

**FIGURE 10:**
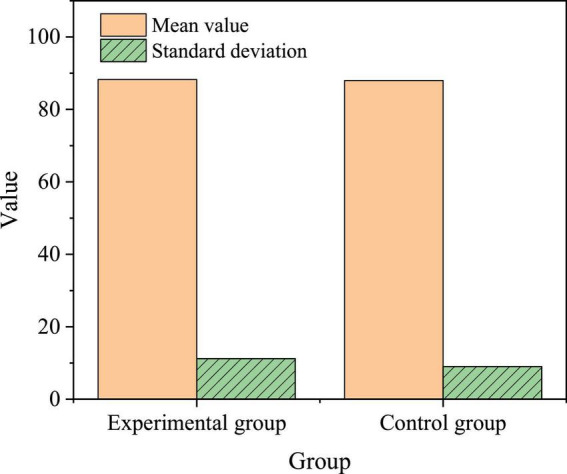
EPL comparison between the experimental group and the control group before the experiment.

Before the experiment, the Students’ EPL is tested in the experimental and control groups. Figure 10 describes the results. Figure 10 implies that the experimental and control groups’ average EPL scores are 88.25 and 87.98, respectively, with a small difference. SPSS21.0 is used in the independent-samples *t*-test. The results show that there is no significant difference between the experimental group and the control group. *t* = 0.042, *p* = 0.970. Hence, the comprehensive EPL of the two groups is on the same level before the experiment.

### Verification results of vocabulary test under the proposed teaching model

**FIGURE 11:**
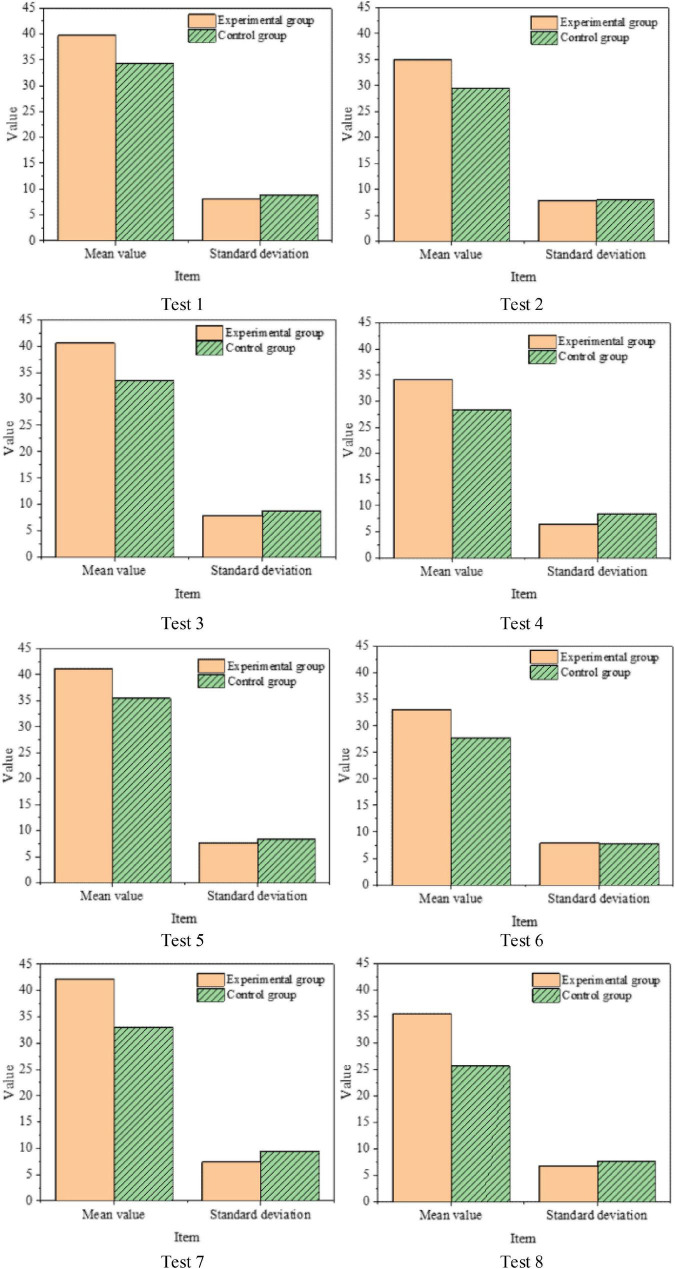
Comparison of vocabulary test scores between experimental group and control group.

Figure 11 displays the results of eight rounds of tests in the experimental and control groups. Figure 11 reveals that the Students’ average scores in the experimental group are higher than those in the control group, so the proposed teaching model can improve Students’ EVM efficiency. SPSS21.0 is used in the independent-samples *t*-test. The results show that the experimental group scored significantly higher than the control group (*p* < 0.05). Thus, the proposed teaching model can improve Students’ EVM efficiency and consolidate their memory.

### Verification results of english proficiency level test under the proposed model

**FIGURE 12:**
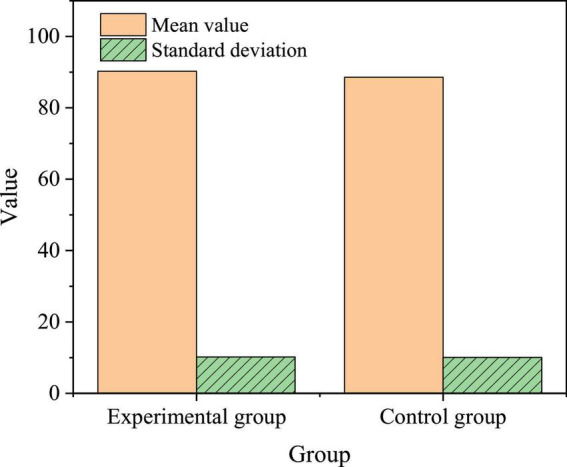
Comparison of final-exam EPL between the experimental group and the control group.

Further, the proposed model’s negative impact on the overall EPL is tested using the final-exam score. Figure 12 manifests the specific results. Figure 12 reveals that the experimental group has scored over 90 points on average, outperforming the control group. SPSS21.0 is used in the independent-samples *t*-test, finding no significant difference (*p* < 0.05). This verifies that the proposed teaching model has no negative effect on the experimental group’s comprehensive EPL.

This work aims to use the PAR method to explore integrating MHE into the college Students’ EVM to help solve some stubborn problems. At the same time, some psychological techniques are gradually infiltrated, such as the cultivation of Students’ learning psychology, the development of memory potential, the cultivation of vocabulary memory habits, and the improvement of vocabulary lifelong learning ability. Finally, the proposed teaching model improves the unfavorable situation of Students’ EVM in teaching practice. The numerical results indicate that the model significantly affects Students’ problem-solving abilities. This work is a successful PAR with many practical achievements worthy of popularization and application. For illustration, it gives educators a more comprehensive and in-depth understanding of school MHE. It forms a typical example of psychological factors playing a positive role in EVM. It provides valuable teaching reflection ideas and scientific research direction for front-line teachers, provides an example for deepening PAR in practice, and constructs a convenient and easy-to-operate English vocabulary teaching model.

## Discussion

The action implementation process proves that this work is a successful PAR. In particular, it integrates the MHE into college Students’ EVM learning link. It improves the neglected practical problems in Students’ EVM practice to a certain extent. The research expectation has been met, and many practical achievements have been gained, worthy of popularization and application. The research extends the definition and content of the MHE infiltration, enabling educators to have a more comprehensive and in-depth understanding of school MHE. At the same time, it demonstrates the role of psychological factors in EVM, explains the necessity of MHE in college Students’ EVM, and makes it more in-depth and specific in school education practice. As such, it widens the way for applying educational psychology and forms a typical example of psychological factors playing a positive role in EVM. Last but not least, this work analyzes and summarizes the problems observed by teachers in the long-term English teaching practice and provides valuable teaching reflections and scientific research direction for front-line teachers. The proposal enlightens teachers on the reasons behind Students’ English learning problems to avoid possible brutal teaching activities and increase rational teaching and learning. The research starts from the main learning body, the current situation of College English teaching, teaching methods, and skills. The analysis and investigation have revealed the fundamental reason behind ineffective vocabulary teaching. For example, the psychological factors in learning, memory theory, and vocabulary memory methods have not been thoroughly implemented in teaching practices. Theories and practices are disconnected. Therefore, the PAR method is chosen to solve this problem and provide a basis for PRA in practice.

The proposed English teaching model based on scientific theory integrates the content of MHE. Meanwhile, it fully exerts Students’ positive psychological factors in EVM and promotes the benign development of English education. Further, it has solved and improved practical teaching problems. The proposed teaching model has passed validity tests, formal school examinations, and QS, opening up a way for front-line teachers’ English vocabulary teaching in CAUs.

The proposed teaching model can directly apply to practical teaching, convenient and easy to operate. It improves Students’ EVM efficiency, consistent with the research results of other scholars (Jiang and Liu, 2020). Moreover, the proposed teaching model only uses the classroom and morning reading time and does not schedule additional memory homework. Without corporal punishment and coercion, it can help students remember words more effectively. Thus, it reduces Students’ schoolwork burden and alleviates their psychological pressure. Such is conducive to the harmony of the teacher-student relationship and Students’ and teachers’ physical and mental health. Furthermore, the teaching mode of “students as the main body and teachers as the guidance” can cultivate college Students’ EVM self-learning ability and lifelong learning habits. Improving Students’ EVM self-learning ability will promote English teaching and the benign development of teaching and learning. In the process of action implementation, the teachers’ feedback verifies once again that with more vocabulary being mastered, teaching and learning become easier. Consequently, in-depth understanding becomes possible, strengthening EVM and, thus, Students’ learning interests. The tacit cooperation between teachers and students speeds up the teaching progress. It makes up for the classroom time in introduction and application mode. In addition, it can complete the semester teaching tasks in advance and win more time to learn additional knowledge. Ultimately, the findings imply that the proposed teaching model has practical value worthy of popularization and application and can help MHE integrate with English teaching. This work did not carry out multi-directional exploration. The follow-up research expects more systematic and comprehensive research and reaches more diverse conclusions.

## Conclusion

School MHE is infiltrated into college Students’ EVM through a PAS to understand the college Students’ EVM performance, teaching behaviors, current situation, and problems. Consequently, combined with relevant theories, a college Students’ EVM-oriented teaching model is proposed, whose effectiveness is verified by teaching experiments. The results show that the experimental group scored significantly higher than the control group (*p* < 0.05). Thus, the proposed model can improve the Students’ EVM efficiency and consolidate their memory. Moreover, the comprehensive EPL of the experimental group is not negatively affected. An important guiding significance is provided for stimulating Students’ interest in English vocabulary learning and improving the quality of vocabulary teaching.

The deficiency is that the teaching experiment is only conducted on 100 students in a college. Therefore, the following questions arise: Can this work’s results reflect the situation of students in other schools? Do they have the same vocabulary learning concepts and strategies? Obviously, the subjects do not suffice to represent all college students. Future research can recruit more students and teachers for a more complete and convincing result. Secondly, this work has not carried out a deep and systematic multi-directional exploration. The follow-up research is hoped to be more systematic and comprehensive to obtain more diverse conclusions.

The following research will explore the definition and content of MHE infiltration so that educators can have a more comprehensive and in-depth understanding of school MHE. Students from different schools and regions can be selected, such as urban and non-urban schools, top universities, and ordinary universities. As such, the research conclusions will be more representative and universal. The follow-up research can also focus on the guidance strategies of vocabulary learning strategies, which will have great practical reference value.

## Data availability statement

The raw data supporting the conclusions of this article will be made available by the authors, without undue reservation.

## Ethics statement

The studies involving human participants were reviewed and approved by the Shaanxi Xueqian Normal University Ethics Committee. The patients/participants provided their written informed consent to participate in this study. Written informed consent was obtained from the individual(s) for the publication of any potentially identifiable images or data included in this article.

## Author contributions

The author confirms being the sole contributor of this work and has approved it for publication.
